# Investigating the atomic behavior of carbon nanotubes as nanopumps

**DOI:** 10.1038/s41598-023-45298-7

**Published:** 2023-10-23

**Authors:** Mehran Shahryari, Akbar Nazari-Golshan, S. Salman Nourazar, Mohsen Abedi

**Affiliations:** 1 Satellite Research Institute, Iranian Space Research Center, Tehran, 1997994313 Iran; 2 Physics Department, Shahed University, 18155/159, Tehran, Iran; 3 Department of Mechanical Engineering, Amirkabir University of Technology, Tehran, 15875-4413 Iran

**Keywords:** Applied physics, Fluid dynamics, Molecular machines and motors, Nanofluidics, Computational methods, Carbon nanotubes and fullerenes

## Abstract

In this study, we utilized molecular dynamics (MD) simulations to investigate the nano pumping process of Carbon Nanotube (CNT) in an aqueous environment. In this research, an attempt has been made to investigate and analyze the pumping process of fullerene C_20_ and water molecules through a carbon nanotube that is externally stimulated by two oscillators. It should be noted that this nano pump is completely immersed in an aqueous environment and the inside and outside of the carbon nanotube is filled with water molecules. To simulate the aqueous environment with NaCl impurities and carbon structures, we employed the Universal Force Field and Tersoff interatomic potentials, respectively. The stability of the simulated structures was demonstrated through an equilibrium process, which was a result of the appropriate settings in our MD simulations. To describe the CNT nano pumping process, we analyzed the velocity and translational/rotational components of C_20_ kinetic energy over time steps. By decreasing the water impurity concentration from 0.50 to 0.075 mol/l, the nano pumping time varied from 10.98 to 10.11 ps, respectively. Additionally, optimization of the atomic wave producing in the nano pumping process led to a further decrease in pumping time to 10.01 ps. Finally, a 2.86% variation in calculated results was observed by changing the water MD simulation model from SPC to TIP4P.

## Introduction

Nanotechnology involves manipulating structures on a very small scale, down to the atomic level. The original definition of this field focused on using this technology to precisely optimize atoms for use in larger applications^1,2^. The National Nanotechnology Initiative defined nanotechnology as the optimization of substances with dimensions ranging from 1 to 100 nm. This broad definition acknowledges the significance of atomic behavior at this scale, resulting in the reclassification of nanotechnology as a research category encompassing all studies on the physical properties of substances that occur on the nano-metric scale. Carbon Nanotube (CNT) is one of the most promising materials for a range of technological applications among different nanostructures^3,4^. To put it simply, carbon nanotubes (CNTs) are tiny tubes made up of carbon atoms with diameters usually measured in the nanometer range. The atomic arrangement of a CNT is a hexagonal pattern arranged in a cylindrical shape, with the carbon atoms at the vertices of the pattern. CNTs have a fixed bond length between carbon atoms which makes them useful for transferring nanometer-scale structures. They are incredibly strong and rigid due to the covalent sp^2^ bonds between carbon atoms, making them the strongest and stiffest structures known in terms of tensile strength and elastic modulus. Because of their environmentally friendly properties, CNTs have potential applications in the field of biomedical technology^5^. To give an example, Carbon Nanotubes (CNTs) have been utilized in drug delivery as a means of nano-sized transportation and have also played a role in the development of a new area of medical science called in situ human organ pressure detection^6,7^. Another application of CNTs is in the process of nano-pumping, which has been introduced in recent years. The attractive forces, known as van der Waals forces, between external atoms and the CNT structure make the atomic process (nano-pumping) feasible. Inserted atoms tend to remain inside the CNT due to these attractive forces, and there is an energy barrier preventing them from escaping^8–11^. To use CNTs for biomedical applications, it is necessary to find a method to effectively remove atoms from the nanotube. In order to achieve this, researchers have investigated various actuation mechanisms. One such mechanism, called "nano pumping," involves generating Rayleigh traveling waves on the surface of the CNT to activate an axial gas flow inside the nanotube, as proposed by Insepov et al.^12^. Chen et al.^13^ have also demonstrated that CNTs can be used to nanopump molecules under different conditions. Other research groups have investigated this behavior of CNTs using various methods^14–17^. Molecular Dynamics (MD) is an important approach used to study the atomic behavior of different materials^18–21^. A special nano pump has been presented for transferring neon gas atoms using the Drexler-Merkel gear system and its performance has been demonstrated by molecular dynamics simulations^22^. This nano pump has been investigated in three different modes (1) pulsed, (2) working with a constant and constant angular velocity and (3) with a constant torque from the stationary state. The proposed nano pump has many challenges for design and manufacturing, and its rotor rotation mechanism is very complicated. The capability and possibility of pumping other heavier molecules by this nano pump have not been investigated. By conducting molecular dynamics simulations, it has been shown that the friction of gas particles and nanotube walls and the creation of travelling Rayleigh waves can be used for the nano pumping of atoms and molecules of hydrogen and helium gas^12^. This is while hydrogen and helium gas molecules are lighter than carbon and the performance of the presented model has only been investigated in vacuum conditions and the effects of surrounding molecules have not been investigated. This idea for the transfer of liquids, especially water molecules, has not yet been evaluated and verified. On the other hand, the number of atoms used in the gas flow simulation is very limited (128–256 atoms) and the gas density is much lower than the real conditions. Using molecular dynamics simulations, it has been shown that through composite nanotubes including surfaces with high and low energy and the application of symmetrical temperature gradients, there is fluid transmission power in the nanochannel^23^. The nanochannel used in this simulation is very different from the carbon nanotube in that its cross-section is rectangular and half of it is made of a high-energy material and the other half is made of a low-energy material. This energy level difference has been applied by applying a symmetrical temperature gradient along the nanochannel (which is very difficult to establish). In the simulation, the bipolar structure of water molecules was not considered, and the effect of long-range Coulombic forces on water molecules was not considered. Using molecular dynamics simulations, the pumping of water molecules through a carbon nanotube under the effect of alternating electric fields (AC) has been investigated^24^. These alternating electric fields are created by the electrodes whose electric charge is fluctuating and are connected to the body of the carbon nanotube. In this research, all carbon nanotube atoms are assumed to be fixed in space and interaction with the atoms of water molecules is not allowed. Also, the effect of carbon nanotube immersion in water molecules and the effect of different water models on the plasticity of momentary dipoles of water molecules and the movement of water molecules, in general, have not been investigated. By conducting molecular dynamics simulations, it has been shown that by using a membrane made of carbon nanotubes with a temperature gradient of $15^\circ \mathrm{C}$ on both sides, water molecules are separated from seawater and transferred to the other side of the membrane^25^. In this method, each carbon nanotube whose two sides have a temperature difference of $15^\circ \mathrm{C}$ acts as a nano pump for water molecules. In this research, it is assumed that the space between the carbon nanotubes is completely blocked and is free of any water molecules and Na+ and Cl− ions. The method of applying the temperature gradient by nanotubes filled with hot and cold water (with a temperature difference of $15^\circ \mathrm{C}$) at a distance of 1.7 nm from carbon nanotubes is very complicated. To analyze the properties of large atomic systems, computational methods such as MD simulations are utilized because they are too complex to estimate analytically due to their vast number of atoms. In this study, the researchers employed the MD method to examine the nano pumping process of CNTs by using Platinum oscillating tips to generate wave propagation in the CNT structure. The researchers were able to demonstrate the capability of the CNT structure to perform nano pumping by transferring a C_20_ molecule through the structure. It has been tried to investigate the performance of the nano pump introduced in this research in different environments and different numerical models for water molecules. Also, by using a large number of atoms in the simulations, the operating environment of the introduced nano pump is very close to reality in terms of water density and pump immersion in the water environment. So the number of water molecules in the simulation box is completely consistent with the number of water molecules with a density of 996.82 kg/m^3^ (the density of water molecules at a temperature of 300 K and 1 bar pressure). The performance of this nano pump in water environments where impurity in the form of dissolved Cl^-^ and Na^+^ ions has also been investigated and even in conventional concentrations of these types of ions.

## Computational method

**Figure 1:**
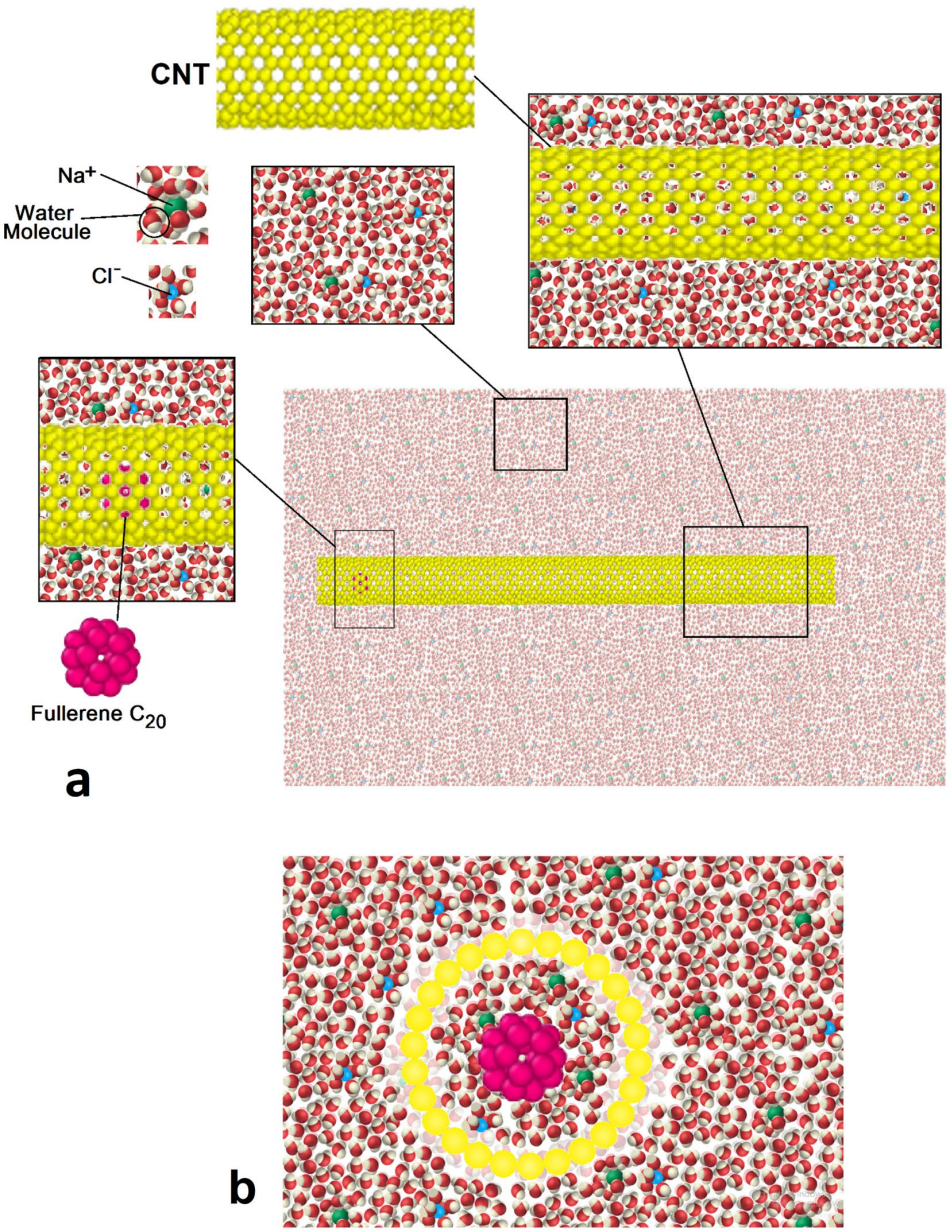
Schematic of atomic configurations arrangement in MD simulation box in (**a**) top and (**b**) side views.

The MD simulations carried out in this study involved the interaction of Pt, C, H, O, Na, and Cl atoms through force fields for a total of 102,000 time steps. Each time step is equal to 1 femtosecond. This value is constant for the time step in all the analyzes performed during the research. This process was modeled using the LAMMPS package^26–29^, which is a Large Scale Atomic Molecular Massively Simulator. In other words, by using this computational package, CNT, H_2_O molecules, and NaCl ions are simulated as Fig. 1. This atomic configuration is shown by Open Visualization Tool (OVITO) software^30^. Computationally, in specified structure, periodic boundary conditions were implemented in x, y, and z directions^31^. In the next step of the simulation algorithm, an NVT ensemble is used to minimize the energy of the simulated system^27,28^. This computational set brings the CNT and the aqueous medium to thermal equilibrium at T = 300 K with a damping rate of 0.1 (Tdamp), for which the simulation is performed in about 1000000-time steps.

The interatomic potential is a key factor in MD simulations. For the current study, Tersoff potentials and Universal Force Field (UFF)^29,32^ are utilized to simulate the atomic system. In UFF potential, atomic behaviors are characterized by bonding and non-bonding terms. Equation 1 defines the non-bonding term between different atoms, which is formulated based on the Lennard–Jones (LJ) relation. This computational relation was originally proposed by John Lennard Jones in 1924^31^, and is presented below:

1 $$ U(r) = 4\varepsilon \left[ {\left( {\frac{\sigma }{{r_{ij} }}} \right)^{12} - \left( {\frac{\sigma }{{r_{ij} }}} \right)^{6} } \right]\quad r \ll r_{c} $$

The equation above describes the relationship between various parameters, including ε which indicates the potential well depth, σ which is the distance at which the particle–particle potential energy is zero $\left( {U\left( \sigma \right) = 0} \right)$, and ***r*** which represents the distance between particles whose distance is less than the cut-off radius (***r***_***c***_). The values of ε and σ depend on the types of atoms present in the MD simulation box, and are determined based on the information provided in Table 1^29^. The values of σ and ϵ in Eq. 1 between similar atoms are precisely what is given in Table 1. But the values of σ and ϵ between dissimilar atoms in Eq. 1 are used from Lorentz-Berthelot combining rules. For example, the non-bonding potential between two hydrogen atoms and two hydrogen and carbon atoms is calculated as follows and with the help of the values given in Table 1 , $\sigma_{HH} = \sigma_{H}$ , $\varepsilon_{HH} = \varepsilon_{H}$ and $\sigma_{HC} = \frac{1}{2}(\sigma_{H} + \sigma_{C} )$ , $\varepsilon_{HC} = (\varepsilon_{H} \varepsilon_{C} )^{\frac{1}{2}}$.

**Table 1:** The ε and σ values associated with LJ interaction for a range of simulated elements^29^.

Element	σ(Å)	ε (kcal/mol)
H	2.886	0.044
O	3.500	0.060
C	3.851	0.105
Na	2.983	0.030
Cl	3.947	0.227
Pt	2.754	0.080

Additionally, we take into account electrostatic interactions between different atoms, and obtain accurate results by utilizing the Coulombic equation.

Technically, H_2_O molecules in our simulations were defined with SPC, TIP3P, and TIP4P models, and finally, the results of them were compared with each other. Further, as stated before, Tersoff potential has been used for C atoms interaction in C_20_ and CNT atomic structures. After defining the initial positions of the particles and specifying the relevant solution parameters in the computational model, the molecular dynamics simulation is carried out in two steps as outlined below:

*Step A* Initial aqueous environment and CNT/C_20_ mixture were simulated with UFF and Tersoff interatomic potentials. In order to study the initial equilibrium state at the beginning of the simulation, by allocating 1,000,000 time steps, the atoms were placed in their stable equilibrium condition according to the limitations of the model and NVT ensemble. For this purpose, the temperature was kept constant at 300 K by using the Nosé–Hoover thermostat so that the system would reach stability at this temperature.

*Step B* Next, the nano pumping process is implemented to equilibrated structures by Pt tips oscillation in the MD simulation box. In order to investigate the nano pumping process, physical parameters such as the speed of C_20_ fullerene as well as the kinetic energy components (translational and rotational) of fullerene were calculated and reported at each time step. The effect of the following parameters was also evaluated :

1. NaCl impurity concentration,
2. amplitude/frequency variation of Pt tips oscillation, and
3. atomic model of H_2_O molecules on CNT nano pumping behavior was reported.

## Results and discussion

### Atomic equilibration of simulated structures

**Figure 2:**
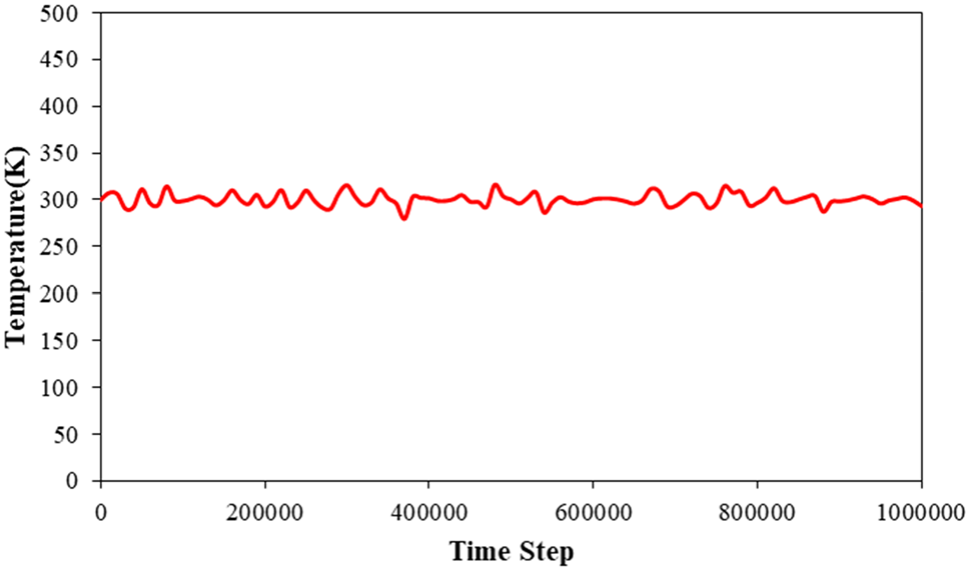
Temperature changes of primary atomic structure as a function of MD simulation time steps.

**Figure 3:**
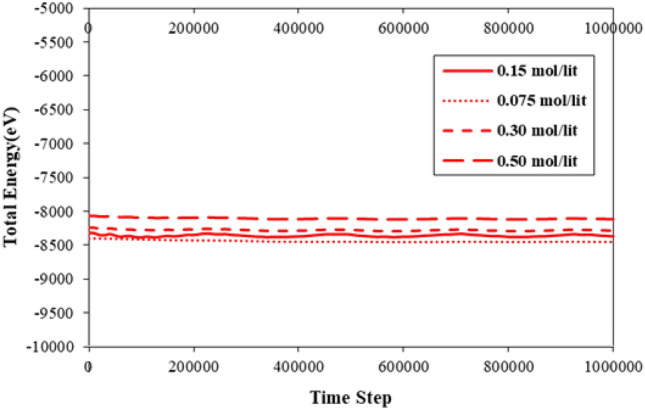
Total energy variation of atomic structures with different NaCl impurity ratios as a function of MD simulation time steps.

The initial step of the simulation involved investigating the atomic behavior of simulated structures such as CNT, C_20_, and a pristine fluid consisting of H_2_O molecules with NaCl impurity, at an initial temperature of T = 300 K. The results obtained during the thermal equilibrium process demonstrate that the position of atoms in the simulated system aligns with the defined interatomic potentials. In the diagrams presented in Figs. 2 and 3, temperature and total energy changes with time steps of calculation are used to show the state of the atomic structure of the model. The temperature changes shown in the diagram of Fig. 2 identify that in the first stage of the simulation, the temperature of the simulated structure has converged to 300 K. Physically, with the placement of atoms in their final location where the system reaches equilibrium and the atomic oscillations are reduced so that the energy of the system reaches its lowest value. Additionally, Fig. 3 illustrates the changes in the total energy of atomic structures at different NaCl impurity ratios as a function of MD simulation time steps. The total energy is the sum of kinetic and potential energies, where the potential energy is inversely proportional to the average distance between the atoms. When the NaCl impurity ratio is increased from to 0.075 l/mol, 0.30 l/mol, and 0.50 l mol in the pristine fluid, the simulated structures remain intact, and the equilibration phase becomes observable after 1,000,000-time steps. Upon further analysis, it can be inferred that the atomic stability of the simulated structures decreases with an increase in NaCl impurity. Numerically, by atomic impurity increasing, the total energy decreases to − 8448 eV, − 8283 eV, and − 8115 eV from − 8370 eV, respectively. This atomic behavior arises from interatomic interaction between H_2_O molecules and NaCl impurity. The atomic modeling method used for H_2_O molecules is another critical parameter in nano pumping process simulation. This parameter changes from TIP4P-BG and TIP4P to TIP3P and SPC, and the total energy of structures decreases to − 8365 eV and − 8391 eV, respectively.

### Nano pumping process in CNT atomic structure

**Figure 4:**
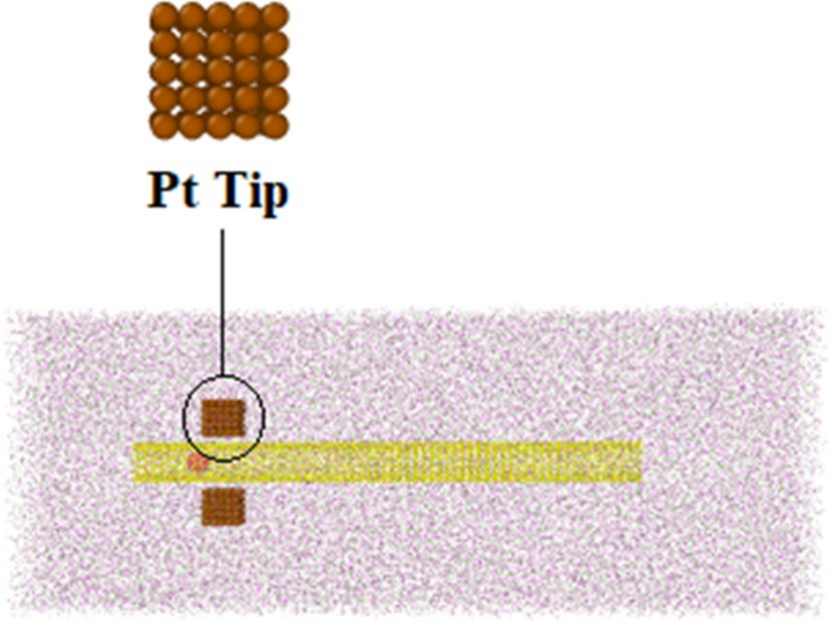
Schematic of atomic structures arrangement in nano pumping process simulation.

**Figure 5:**
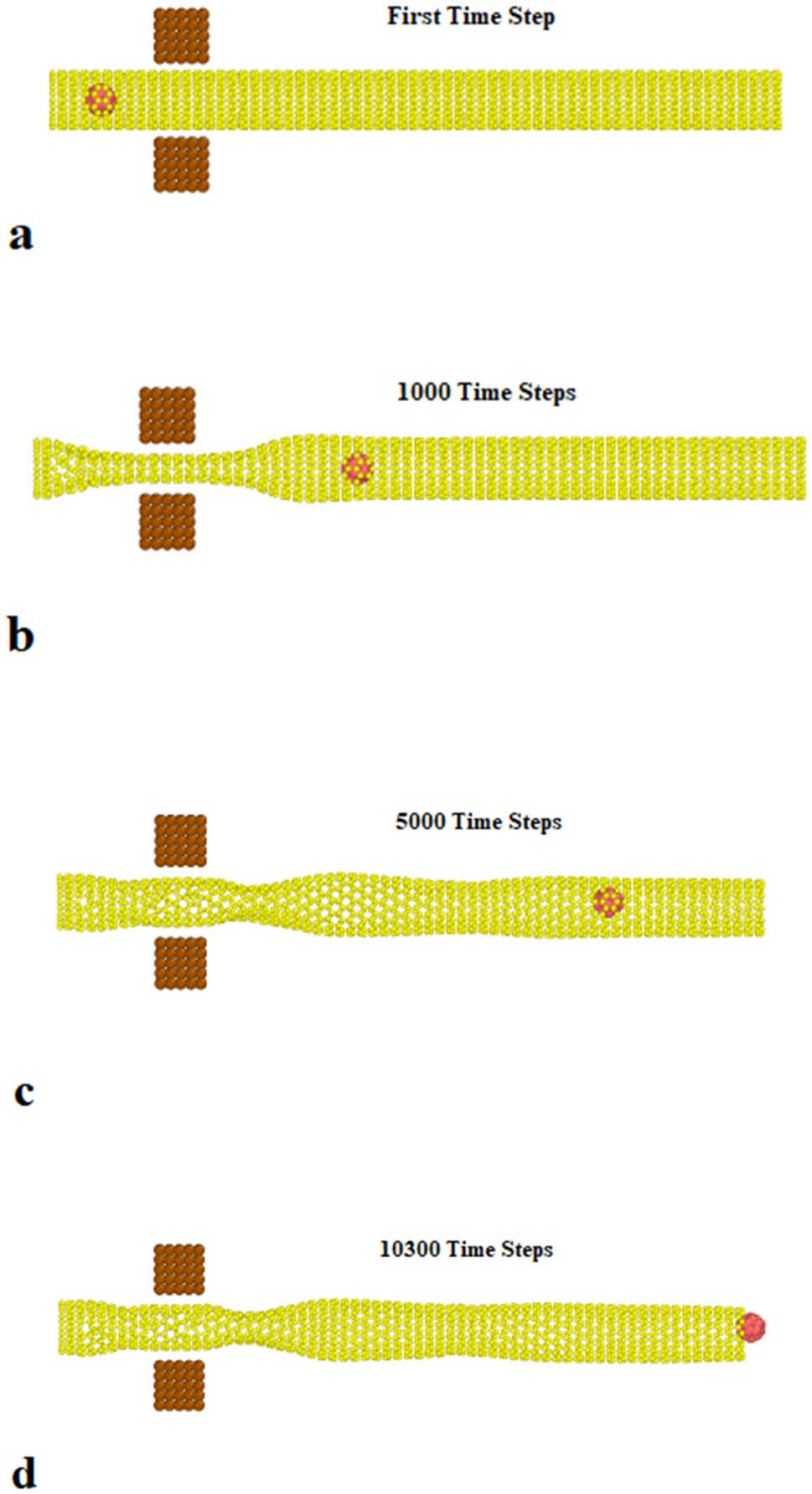
Time evolution of nano pumping process with CNT atomic structure at (**a**) t = 0, (**b**) t = 1000, (**c**) t = 5000, and (**d**) t = 10,300 time steps.

To simulate the pumping process, two platinum tips are added to the initial atomic configuration, shown in Fig. 4. The C_20_ molecule’s speed and kinetic energy are calculated to describe the process of nano pumping inside CNT. The C_20_ fullerene molecule is located in the centerline 50 angstroms ahead of the head of the nanotube. Two oscillating tips are placed in front of each other at a distance of 30 angstroms from the C_20_ fullerene molecule. Two oscillator tips containing 10 × 5 × 5 platinum atoms are located in a simple cubic lattice with a lattice constant of 2 angstroms. The tips oscillate along the carbon nanotube wall to propagate a standing mechanical wave that can eject the trapped molecule from the other end of the nanotube. The position of platinum atoms in the FCC lattice is considered to be relatively constant.The time evolution of the simulated structures is presented in Fig. 5 after 20,000-time steps. The atomic behavior arises from the oscillation of Pt tips with a frequency of 0.75 THz and a magnitude of 2 Å, as described in following equations

2 $$ {\text{y}}_{{{\text{top}}}} = {\text{ Asin}}\left( {{2}\pi {\text{ft}}} \right) $$

3 $$ {\text{y}}_{{{\text{down}}}} = - {\text{Asin}}\left( {{2}\pi {\text{ft}}} \right) $$

The fullerene molecule is pushed by the tip-excited wave and moves in the z direction due to repulsive interaction with the CNT structure. The simulations show that the C_20_ molecule moves from 50 to 136 Å in the z direction after 10,300 time steps. This behavior is consistent with previous reports, indicating the accuracy of the presented MD simulation method^33^.

**Figure 6:**
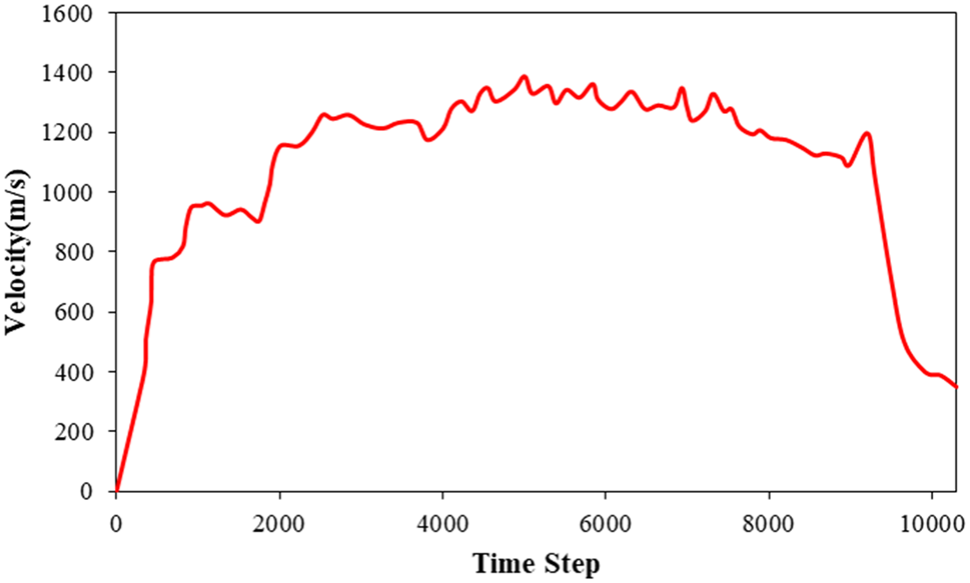
The velocity of the C_20_ molecule as a function of MD simulation time steps during the nano pumping process.

**Figure 7:**
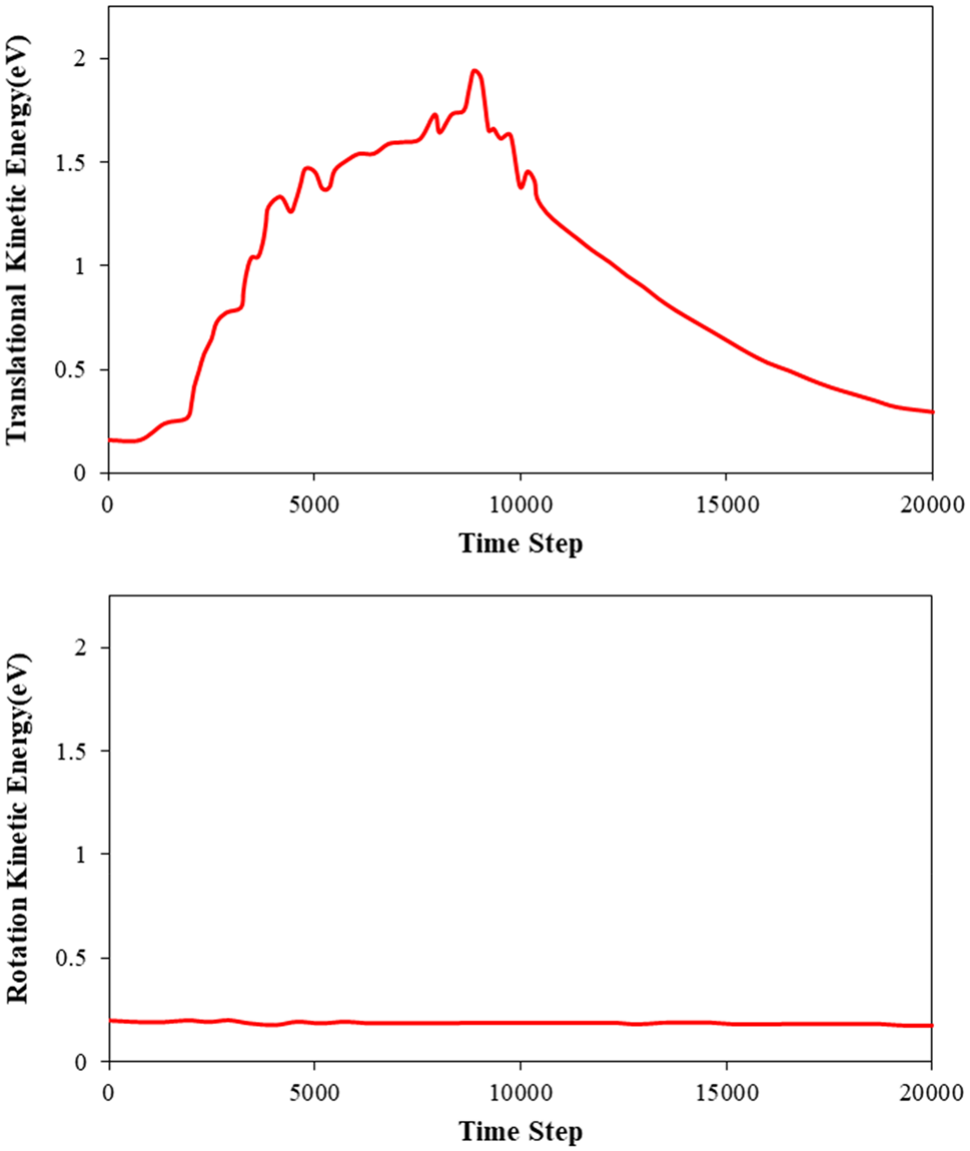
Comparison of (**a**) translational and (**b**) rotational components of C_20_ kinetic energy in the atomic pumping process.

The outcomes reveal that the time steps of the MD simulation are of sufficient length to identify the nanopump-pump operation. The C_20_ molecule’s velocity and kinetic energy during this simulation stage are shown in Figs. 6 and 7, respectively. The C_20_ molecule’s velocity suggests that atomic waves are produced and maintained throughout the MD simulation, increasing the C_20_ molecule’s speed and causing it to move in the direction of wave propagation inside the CNT. Calculations show that the C_20_ molecule reaches the other end of the CNT structure after 9.2 s. At the end of the simulation time, the atomic ball was removed from the CNT, this process lasted for 10.3 s. From a physical point of view, the transfer process (pumping) includes two basic steps to accelerate the C_20_ fullerene molecule in the CNT channel Fig. 6 shows the acceleration and deceleration of the C_20_ molecule as a function of the time steps of the simulation. The graph given in this figure describes that the maximum value of the acceleration of the C_20_ molecule occurs in time steps 0 to 851, resulting in a speed of this molecule of 948 m/s. After this initial phase, the C_20_ molecule increases its speed but at a lower rate so that it reaches a speed of 1361 m/s for the next 4989 time steps. The subsequent phase involved a gradual reduction in the velocity of the C_20_ molecule until it uniformly decelerated to an axial velocity of 1196 m/s and was ejected out of the right-hand end of the CNT structure. The ejection process involved a rapid increase and subsequent decrease in the speed of the C_20_ molecule, which was caused by the interatomic bonds between the fullerene and CNT atoms. Despite the decrease in speed, the C_20_ molecule retained sufficient kinetic energy to overcome the potential energy barrier of the CNT atoms. The numerical analysis showed that the ejection speed of the C_20_ molecule from the CNT structure was 350 m/s, which is a significant physical value for the design of biomedical applications.

Also, the numerical simulation results show that the C_20_ fullerene molecule has a rotational motion during pumping, and in other words, the atomic ball has two translational and rotational motions. The wave inside the carbon nanotube transfers the translational/rotational kinetic energy to the C_20_ molecule through the interaction between C_20_ molecule and CNT atoms (see Fig. 7). Upon analyzing the results more thoroughly, it can be observed that the rotational movement of the fullerene molecule results in the loss of pumping energy. The high-speed rotation of the fullerene molecule disrupts its translational motion along the CNT, leading to a decrease in its translational velocity. Therefore, it is important in biomedical and drug delivery applications to minimize the target material’s rotational velocity to increase translational energy and increase the nano pumping efficiency from this procedure.

Entropy is one of the most important thermodynamic characteristics of a system. Entropy is a measure of the randomness of the system. The system investigated in this research has translational, rotational and vibrational degrees of freedom. Therefore, it has entropy components associated with each of the above DOFs (S_trans, S_rot, S_vib). In addition, it has structural entropy that results from having different bonding and molecular compositions.

In order to further investigate the nanopump process of the fullerene molecule from a thermodynamic point of view, the entropy of the CNT-C_20_ system was investigated.

To calculate the entropy of the fullerene C_20_ molecule, a new fingerprint has been introduced to distinguish between liquid atomic environments of water molecules and solid carbon molecules in the CNT and fullerene C_20_ lattices. In the LAMMPS computational code, this fingerprint is based on an approximate expression for the predicted entropy on individual atoms. When combined with local enthalpy, this fingerprint achieves even finer resolution and can distinguish between crystal structures. The advantage of this parameter over other parameters is that no a priori information about the fullerene structure is required.

This parameter is calculated for atom $i$ using the following formula^34,35^,

4 $$ s_{S}^{i} = - 2\pi \rho k_{B} \mathop \smallint \limits_{0}^{{r_{m} }} \left[ {g\left( r \right)lng\left( r \right) - g\left( r \right) + 1} \right]r^{2} dr $$

where *r* is a distance, *g*(*r*) is the radial distribution function of atom *i* and *ρ* is the system’s density. Since *g*(*r*) calculated for each atom i may have noise, the following equation is used to reduce *g*(*r*) noise:

5 $$ g_{m}^{i} \left( r \right) = \frac{1}{{4\pi \rho r^{2} }}\mathop \sum \limits_{j} \frac{1}{{\sqrt {2\pi \sigma^{2} } }}e^{{ - (r - r_{ij} )^{2} /\left( {2\sigma^{2} } \right)}} $$

This sum is applied to $j$ atoms neighbouring $i$ atom and in this expression, *σ* is a parameter for smoothing control.

In order to average the entropy, we use the following relationship for $j$ atoms around $i$ atom.

6 $$ s_{S}^{i} = \frac{{\mathop \sum \nolimits_{j} s_{S}^{j} + s_{S}^{i} }}{N + 1} $$

**Figure 8:**
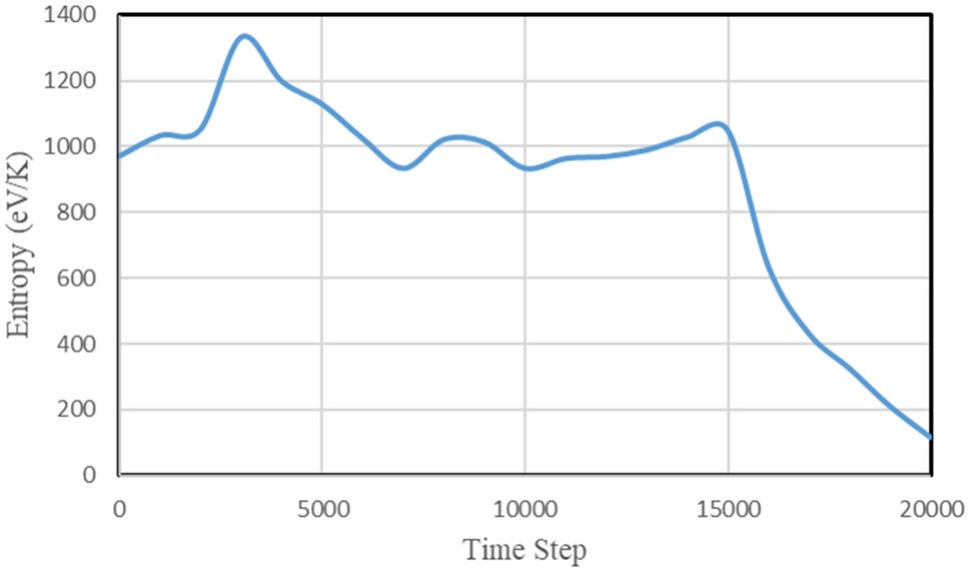
Change in entropy of C_20_ molecule by time step.

The change in the entropy of the C_20_ molecule according to the time step of the fullerene molecule pumping process is shown in Fig. 8.

When the fullerene pumping process starts, the entropy first increases, but with time, it slowly reaches a nearly constant value along the length of the carbon nanotube, but with fluctuations. During the fullerene is leaving the carbon nanotube, the entropy increases, when the fullerene is completely separated from the nanotube and immersed in the aqueous medium, the entropy decreases drastically and converges to 115 eV/K converges.

**Figure 9:**
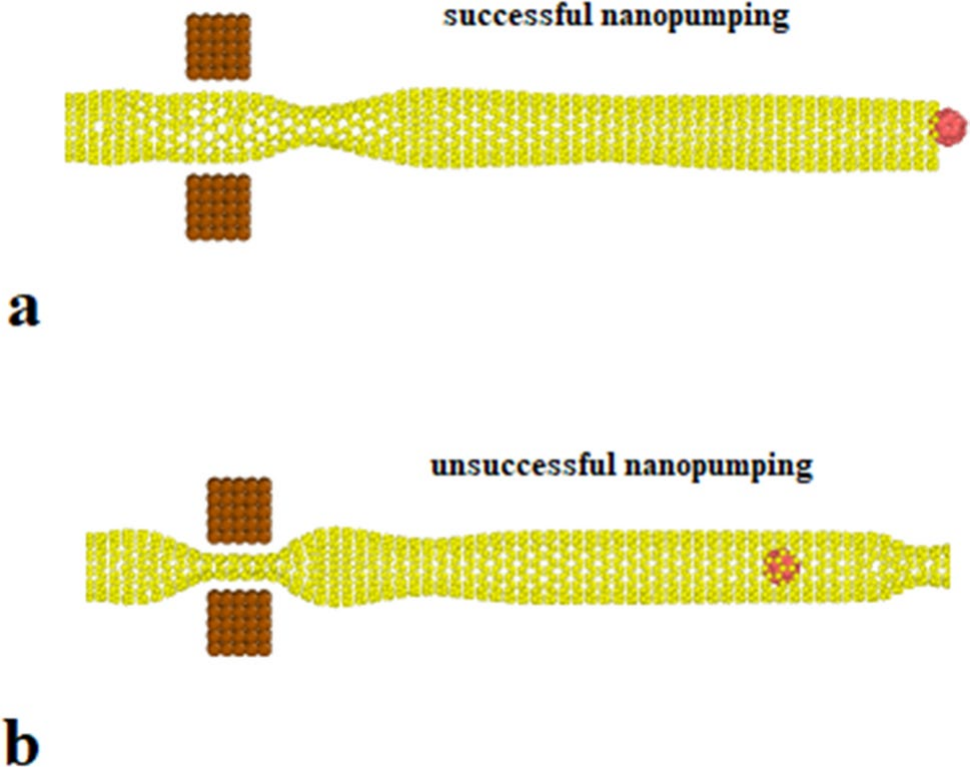
CNT and C_20_ atomic structures positions in the final step of MD simulations in the presence of H_2_O molecules with (**a**) 0.15 mol/l and (**b**) 0.50 mol/l impurity.

The atomic ratio of NaCl impurity is another investigated parameterin the nano pumping process. To represent this atomic parameter effects on the CNT nano pumping procedure, the impurity ratio in pristine H_2_O molecules set to 0.075, 0.15 (neutral value), 0.30, and 0.50 mol/l values. Theoretically, we can describe the effectiveness of nano pumping in these simulation settings by analyzing the energy transfer process in terms of translational and rotational components of kinetic energy. Figure 9 shows a comparison of these two components of C_20_ molecule kinetic energy in the presence of 0.50 mol/l impurity. Our computational results show that the nano pumping process disrupted with 0.5 mol/l impurity value. Further, by NaCl impurity value decreasing to 0.30 mol/l, the nano pumping process detectable in more MD simulation time steps rather than other atomic samples (H_2_O molecules with 0.075 mol/l and 0.15 mol/l impurity). So we can say the atomic impurity decreases the nano pumping process efficiency. Table 2 shows the numerical results of MD simulation at this stage of our study.

**Table 2:** The maximum rate of translational velocity and translational/rotational kinetic energy of the C_20_ molecule as a function of NaCl impurity concentration.

NaCl concentration (mol/l)	Max. velocity (m/s)	Max. transitional energy (eV)	Max. rotational energy (eV)
0.075	1374	1.97	0.19
0.15 (neutral)	1361	1.94	0.20
0.30	1352	1.92	0.23
0.50	1331	1.88	0.27

By simultaneously examining the graphs presented in Figs. 7 and 8, we can conclude that at the beginning of the fullerene pumping process, despite the fact that the kinetic energy of the fullerene molecule is still not high, its entropy is the highest. This issue shows the high influence of oscillators on C_20_ fullerene atoms, which caused the collision and release of energy in the fullerene structure and changed its configuration from spherical.

Halfway and with the acceleration of the fullerene inside the carbon nanotube, which is accompanied by an increase in its energy of motion, the entropy decreases and almost as long as the fullerene ball is inside the carbon nanotube, the entropy changes with relatively small fluctuations. This issue indicates the deformation of the fullerene ball caused by the propagation of mechanical waves in the body of the carbon nanotube, and its effect appears on the body of the C_20_ carbon ball through intermolecular forces.

When the fullerene is removed from the carbon nanotube, due to which it moves away from the force field of the carbon atoms of the nanotube the distortion in the fullerene structure reaches its minimum value, and the entropy also decreases a lot.

Although the increase in the ratio of impurity in the solution causes a relative increase in the rotational kinetic energy of fullerene, it does not have a great effect on the speed and translational kinetic energy of fullerene, so that with an increase of 6.7 times the ratio of impurity in the water solution, the speed only decreases by 3.1% and the translational kinetic energy decreases by about 4.6%.

**Figure 10:**
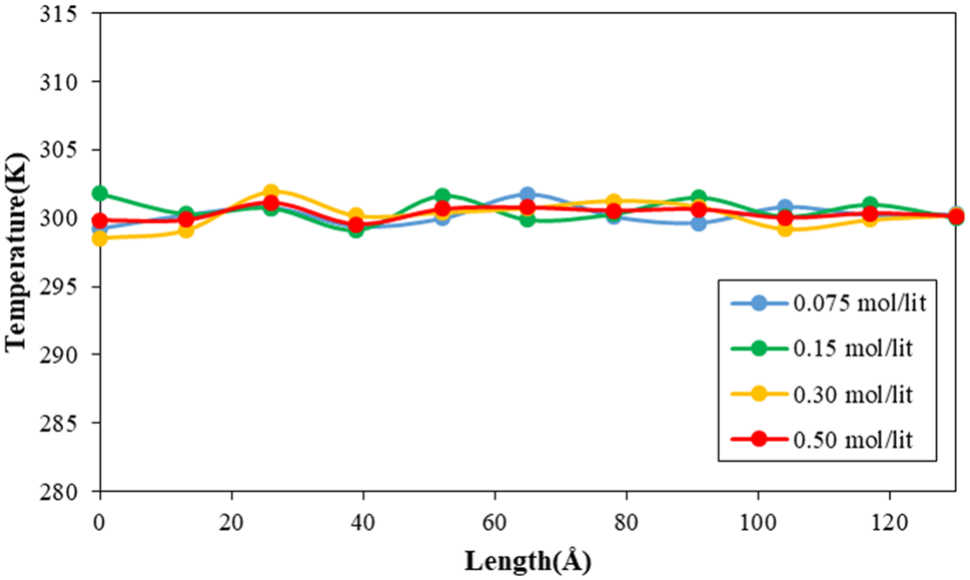
The temperature variation of simulated fluid inside CNT nanostructure as a function of atomic impurity ratio.

**Figure 11:**
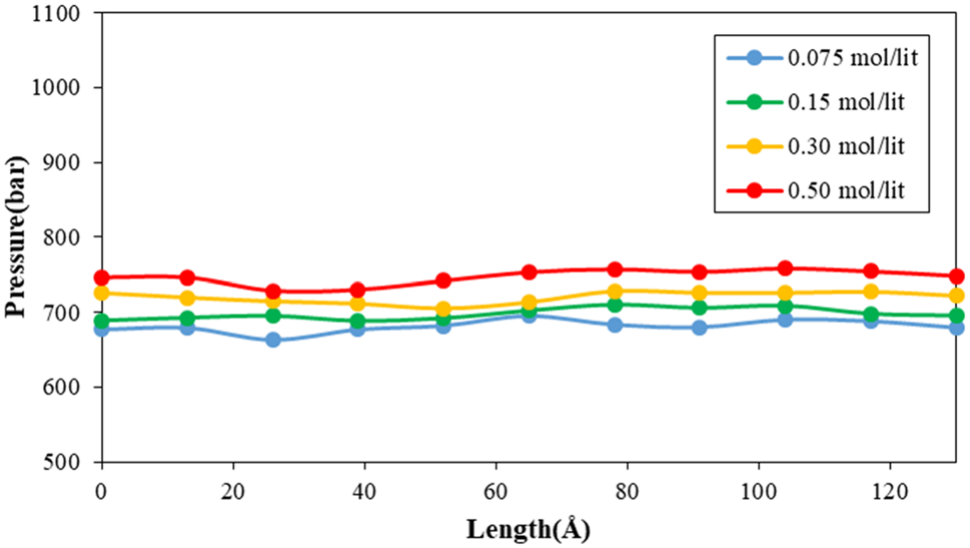
The pressure variation of simulated fluid inside CNT nanostructure as a function of atomic impurity ratio.

In order to depict how fluid molecules behave within a simulated nanotube, the temperature and pressure of these molecules are determined. Figure 10 illustrates how the temperature changes for fluid particles depending on the length of the CNT. As demonstrated in the figure, the temperature of the fluid fluctuates around T = 300 K, indicating the stability of the atoms in all regions of the MD box. A similar outcome is obtained for pressure calculation. As shown in Fig. 11, the pressure distribution does not change significantly in various regions inside the CNT. Furthermore, MD simulations show the atomic pressure ratio increases by adding Na and Cl ions to pristine fluid (H_2_O molecules). Numerically, the maximum ratio of pressure increases from 695.10 to 757.89 bar by ion density enlarging from 0.075 to 0.500 mol/l, respectively. This behavior arises from atomic interaction increases between H_2_O molecules and defined ions.

**Figure 12:**
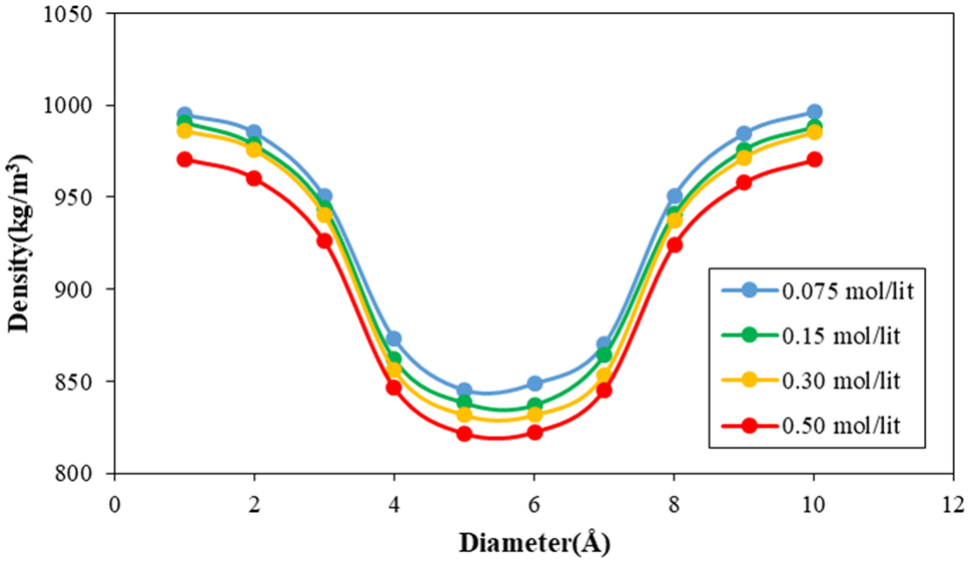
The density of fluid molecules inside CNT nanostructure as a function of atomic impurity ratio.

The density of H_2_O molecules inside CNT can be affected the nano pumping process. This section of our MD study describes H_2_O molecules arrangement by fluid density profile calculation as a radius of the atomic nanotube. As depicted in Fig. 12, the atomic density in the vicinity of CNT increased significantly. This atomic behavior arises from the attraction force implemented on water molecules from carbon atoms. This Van der Waals interaction causes more fluid molecules accumulation near the nanotube wall. Furthermore, by increasing the impurity ratio in the MD simulation box, the density of water molecules increases in the simulation procedure. This atomic evolution shows the impurity ions diffusion inside the CNT nanostructure. Figure 12 shows the in-tube density changes of less than 3% due to impurity changes. Although the increase in impurity causes a decrease in the density of water inside the nanotube, this decrease can be considered insignificant. Numerically, the maximum value of fluid molecules density converged to 970.85 kg/m^3^ value by atomic impurity increasing to 0.50 mol/l.

**Figure 13:**
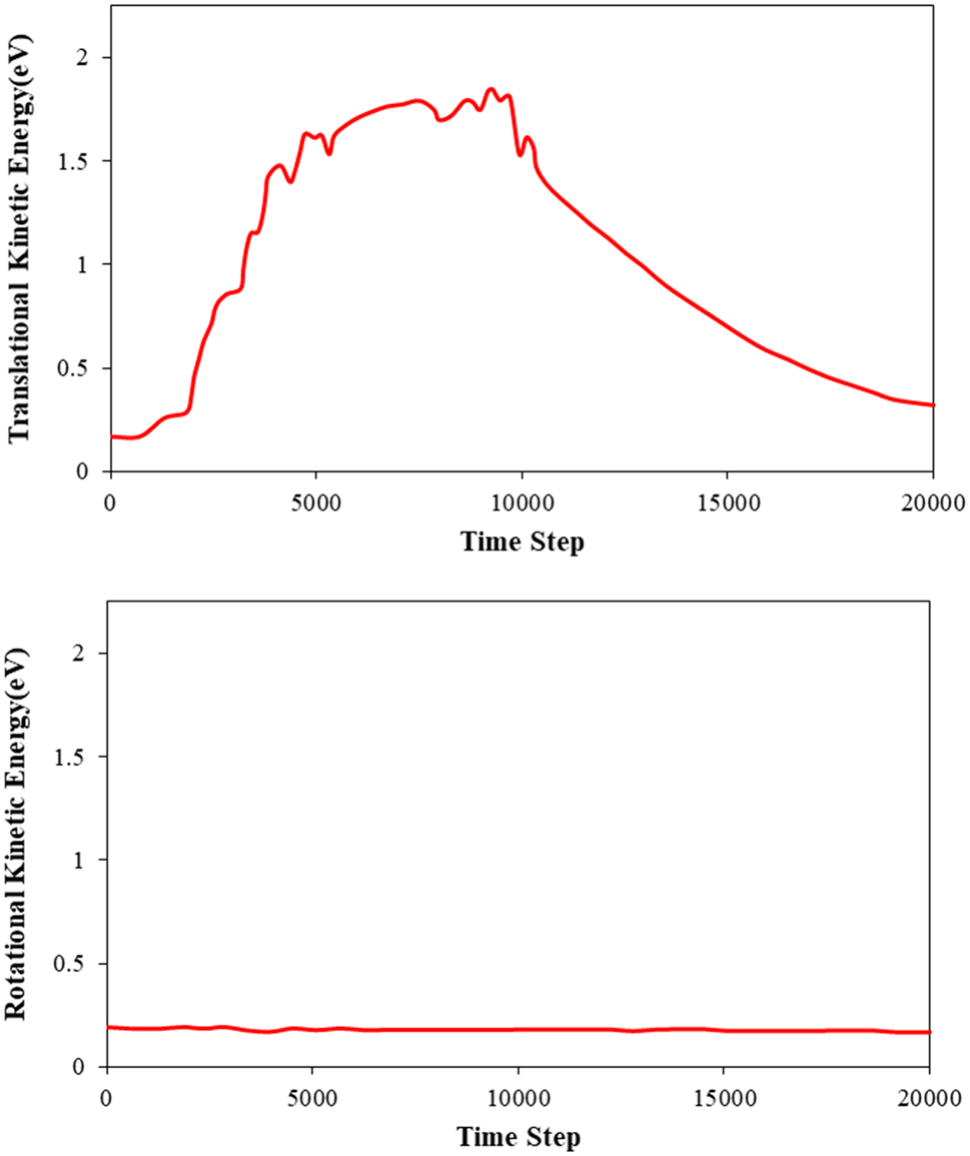
Comparison of kinetic energy partition between the (**a**) translational and (**b**) rotational motion of C_20_ molecule in an optimized nano pumping process.

The nanopumping efficiency can be optimized by manipulating the frequency of the upper and lower Pt tips, in this regard, the simulations show that in the oscillation range of 1.75 Å the C_20_ fullerene pumping inside for (13, 0) CNT is at optimum state. Calculations show that if the amplitude of tip oscillation is larger than 3 Å, the upper region of CNT and the lower region of CNT are very close to each other to the point where the carbon atoms at the top and bottom of CNT form bonds and the oscillation of the platinum tip can no longer excite the carbon ball. Also, by changing the oscillation frequency of the platinum tips, it was determined that the optimal value for the platinum tip oscillation frequency is 0.60 THz. On the other hand, if the oscillation frequency of the platinum tips becomes less than 0.50 THz, the contribution of the rotational kinetic energy of the C_20_ fullerene molecule from The translational kinetic energy is increased so that the molecule just rotates without translational motion. If the frequency of the platinum tips’ oscillation is higher than 2 THz, then there won’t be a significant mechanical wave generated in the CNT structure, and thus the nanopumping process won’t occur. Based on the analysis above, it can be inferred that the optimal values for the C_20_ fullerene molecule pumping process in a (13, 0) CNT structure are an amplitude of 1.75 Å and a frequency of 0.60 THz for the oscillation of the platinum tip. Figure 13 illustrates this optimal process.

**Figure 14:**
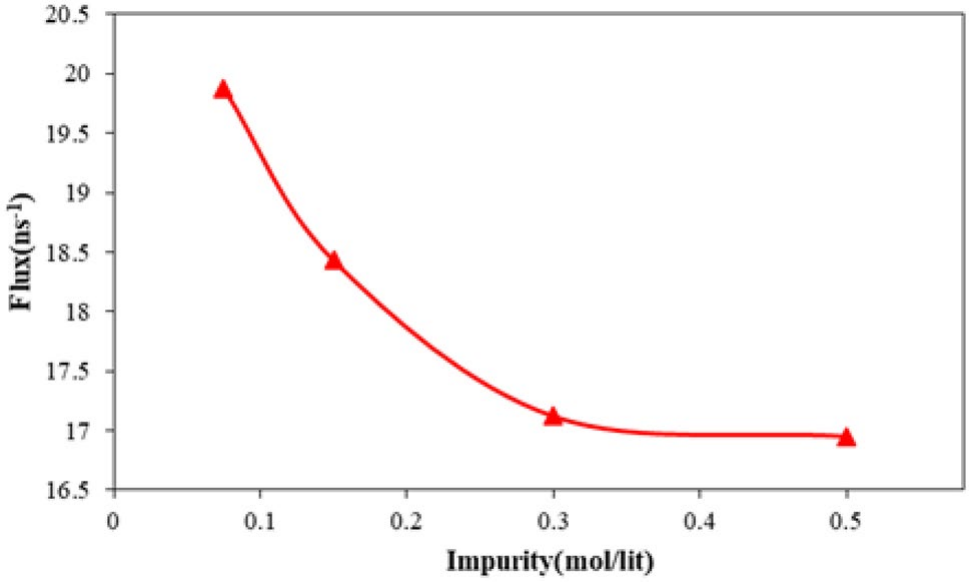
The net flux of H_2_O molecules inside CNT nanostructure as a function of atomic impurity ratio.

**Figure 15:**
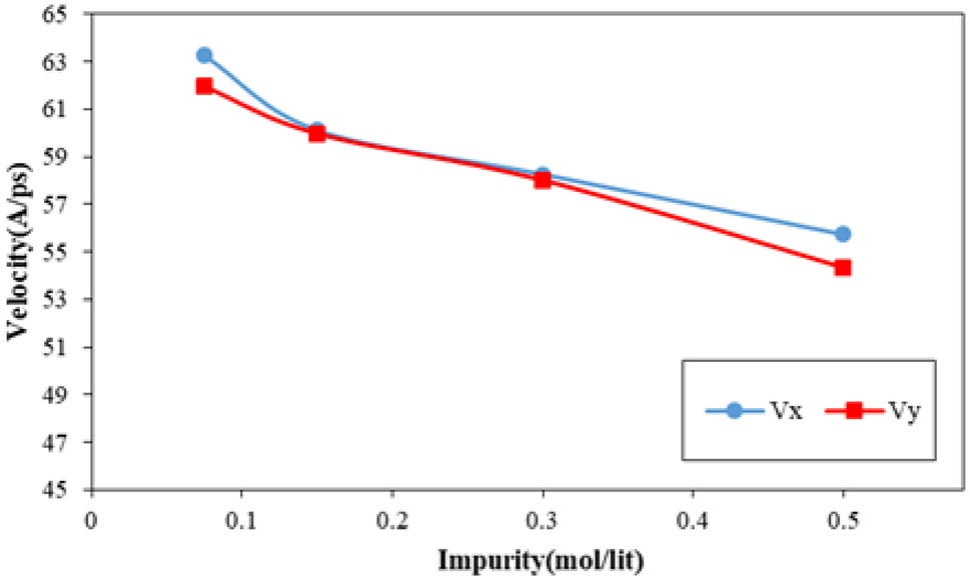
The x and y components of H_2_O molecules velocity inside CNT nanostructure as a function of atomic impurity ratio.

The flux of fluid molecules passed from CNT nanostructure can be described as the nano pumping efficiency in our computational study. Figure 14 shows the flux of simulated fluid as an atomic impurity ratio in the MD box. As depicted in this figure, by atomic impurity increasing in pristine fluid, this atomic section’s flux decreases. This reduction decreased by impurity enlarging in the MD box. Physically, flux reduction in this step of our MD simulations arises from two important cases. Firstly, repulsive interaction between H_2_O molecules in our MD simulations is an important parameter. Secondary, spatial constraints created by atomic impurities for water molecules are another case. Numerically, in simulated structures, the water molecules flux varies from 19.88 to 16.95 ns^−1^ in the nano pumping process. The x and y components of these molecules’ velocity can describe the stated phenomenon properly. As shown in Fig. 15, the velocity component of H_2_O molecules decreases from 63.25 Å/ps to 54.33 Å/ps by atomic impurity increasing from 0.075 to 0.500 mol/l, respectively. So, this parameter reduction shows the atomic displacement limit in defined MD time.

Hong-Fei et al.^36^ applied machine learning and performed multiple molecular dynamics simulations for pure water and used a back-propagation neural network to create an efficient mapping between model parameters and four critical physical properties of water. Finally, they optimized the four-site water model by optimizing the genetic algorithm method with a high population and proposed two new four-site water models. The first model (TIP4P-BG), is a typical four-site water model, and the second model (TIP4P-BGT) is an advanced model with temperature-dependent parameters. Since in the simulations carried out in this research, the NVT ensemble with Nosé-Hoover thermostat was used to keep the temperature of the system constant at 300 K, it is not necessary to use the TIP4P-BGT model for water molecules and the TIP4P-BG model is sufficient. Is. Compared to the TIP4P model, all the parameters except the four parameters q, d, σ and ɛ, the rest of the parameters have remained constant. Table 3 shows these changes. To check the sensitivity of fullerene nano-pumping results and water molecules to the selected model for water molecules, the TIP4P-BG model was also used to simulate the nano-pumping process, and the results are added in Table 4.

**Table 3:** The parameters of the TIP4P and TIP4P-BG water model ^36^.

Parameters	H charge (e)	OM distance (Å)	LJ σ of OO (Å)	ɛ LJ of OO (kcal/mole)
TIP4P	0.520	0.15	3.1536	0.1550
TIP4P-BG	0.51244	0.12097	3.16399	0.14585

**Table 4:** The maximum rate of translational velocity and translational/rotational kinetic energy of the C_20_ molecule as a function of H_2_O models.

H_2_O molecules simulation model	Max. velocity (m/s)	Transitional energy (eV)	Rotational energy (eV)
SPC	1322	1.43	0.28
TIP3P	1333	1.62	0.25
TPP4P	1361	1.94	0.20
TIP4P-BG	1363	1.95	0.19

In our computational study’s final step, we use SPC, TIP3P, and TIP4P models to simulate the H_2_O molecules in the MD simulation box. In this section of the study, we report the maximum velocity, the transitional, and rotational kinetic energy of the C_20_ molecule in the nano pumping process for comparing of stated models results. From reported values in Table 3, we can conclude the model of the H_2_O molecule has not an impressive effect on the nano pumping process. Numerically, the maximum rate of velocity (1361 m/s) calculated with the TIP4P model and the minimum rate estimated for SPC one (1322 m/s). For translational kinetic energy, a similar result is obtained, so we conclude that the nano pumping process will be available by these three atomic models. In other words, by good condition settings for nano pumping procedures such as appropriate impurity rate and oscillation magnitude/frequency, the successful nano pumping process can be detectable for various water molecule models.

## Conclusion

This study used molecular dynamics to examine the behavior of a carbon nanopump under various conditions. Specifically, the movement of a C_20_ molecule in an aqueous medium was analyzed. Overall, the computational findings from the numerical simulations are as follows:

A. Tersoff and UFF interatomic potentials are suitable force fields for molecular dynamics simulation of C_20_ fullerene molecule pumping process in CNT.
B. Although with the increase of NaCl impurity concentration from 0.075 mol/l to 0.50 mol/l, the process of nano-pumping takes longer, this increase is not significant.
C. The oscillation of the platinum tip with amplitude A = 1.75 Å and frequency f = 0.60 THz is the optimal value for the pumping process of C_20_ fullerene molecule in CNT (13, 0) structure.
D. If the frequency of the atomic tip is lower than 0.50 THz, it causes a high rotation of the fullerene molecule, which reduces the translational motion and makes it not pumping.
E. Atomic tips amplitude changes can be disrupted of nano pumping procedure for A > 3 Å.
F. The water molecules’ net flux varies from 19.88 to 16.95 ns^−1^ in the nano pumping process by atomic impurity increasing from 0.075 mol/l to 0.500 mol/l, respectively.
G. SPC, TIP3P, and TIP4P models can be appropriately described of H_2_O molecules effect on CNT nano pumping procedure.

The results obtained from the MD simulations indicate that the pumping process of C_20_ fullerene molecule in CNT is affected by physical parameters such as ion concentration, as well as the amplitude and frequency of oscillation of atomic tips. By adjusting these parameters, it is possible to increase the efficiency of the pumping process.

## Data Availability

The datasets generated and/or analysed during the current study are not publicly available due we do not have consent from all authors to publish the raw data but are available from the corresponding author on reasonable request.

## List of symbols

F_ij_: Interatomic force between atoms i and j (eV/Å)
V_ij_: Interatomic potential (eV)
m: Atom mass (u)
r_c_: Cut-off distance (Å)
r_ij_: Distance between atom i and j (Å)
t: Simulation time (fs)
v: Velocity of atom (Å/ps)
f: Frequency of atomic oscillation
A: Amplitude of atomic oscillation

## Greek symbols

ε: Energy parameter in Lennard–Jones interatomic potential
σ: Length parameter of Lennard–Jones interatomic
Δt: Molecular dynamics time step
